# Supplementary material for: Association between the frequency of television watching and overweight and obesity among women of reproductive age in Nepal: Analysis of data from the Nepal Demographic and Health Survey 2016
Source: PLoS One. 2020 Feb 10;15(2):e0228862. doi: 10.1371/journal.pone.0228862 (PMC7010261; doi:10.1371/journal.pone.0228862)
Supplement: S1 File — (PDF) [file pone.0228862.s001.pdf]

Supplementary Tables

Supplementary Table 1: Crude and Adjusted odds ratios for factors associated with overweight and obesity compared to normal weight among women of Nepal, NDHS 2016.

Variable	Model 1			Model 2			Model 3		
	OR	Lower Limit	Upper Limit	OR	Lower Limit	Upper Limit	OR	Lower Limit	Upper Limit
**Age Group (in years)**									
15-24	Ref						Ref		
25-34	3.8***	3.3	4.5				2.3***	1.9	2.8
35-49	5.6***	4.8	6.5				3.7***	3.0	4.6
**Place of Residence**									
Rural	Ref			Ref			Ref		
Urban	1.6***	1.3	2.0	1.1[1]	0.9	1.4	1.1	0.9	1.4
**Province of Residence**									
Province 1	Ref						Ref		
Province 2	0.4***	0.3	0.5				0.4***	0.3	0.5
Province 3	1.4*	1.1	1.9				1.2	0.9	1.5
Province 4	1.3[1]	0.9	1.7				1.2	0.9	1.5
Province 5	0.7*	0.5	0.9				0.7**	0.6	0.9
Province 6	0.5***	0.3	0.6				0.6***	0.5	0.8
Province 7	0.3***	0.2	0.4				0.4***	0.3	0.5
**Ecological Zone of Residence**									
Mountain	Ref						Ref		
Hill	1.5*	1.0	2.2				0.8	0.7	1.1
The Terai	1.1	0.7	1.6				0.7*	0.5	0.9
**Highest Educational**									

**Status**									
No Formal Education	Ref						Ref		
Primary	1.3*	1.1	1.5				1.6***	1.3	1.9
Secondary	0.6***	0.6	0.7				1.4***	1.2	1.7
Higher	0.6***	0.5	0.7				1.2***	0.9	1.5
**Currently employment**									
No	Ref						Ref		
Yes	1.3	1.2	1.5				1.0	0.8	1.1
**Wealth index**									
Poorest	Ref						Ref		
Poorer	1.4**	1.1	1.7				1.4**	1.1	1.7
Middle	1.5***	1.2	1.9				1.6***	1.2	1.9
Richer	2.3***	1.9	2.9				2.1***	1.7	2.7
Rich	5.1***	4.1	6.4				4.5***	3.5	5.8
**Marital Status**									
Single	Ref						Ref		
Married	5.1***	4.3	6.1				2.1***	1.6	2.8
Separated/ Divorced/ Widowed	4.3***	3.0	6.2				1.6*	1.0	2.4
**Parity**									
0	Ref						Ref		
1	2.7***	2.2	3.3				1.1	0.8	1.5
2	5.2***	4.4	6.3				1.4*	1.1	1.8
3	5.4***	4.4	6.6				1.4*	1.0	1.9
3+	4.2***	3.5	5.1				1.1	0.8	1.6
**Number of Household Member**									
≤5	Ref						Ref		
>5	0.7***	0.6	0.8				0.9	0.8	1.0
**Frequency of watching TV**									
Not at all	Ref			Ref			Ref		

Less than once a week	1.1	0.9	1.3	1.2[1]	0.9	1.5	1.2	0.9	1.5
At least once a week	1.7***	1.5	2.0	1.8***	1.5	2.3	1.3*	1.0	1.7
**Interaction Between Place of Residence with Frequency of Watching TV**									
Urban Residence × Not at all watches TV				Ref			Ref		
Urban Residence × Watches TV less than once a week				1.0	0.7	1.4	0.9	0.6	1.2
Urban Residence × Watches TV at least once a week				1.3*	1.0	1.8	1.0	0.7	1.3

NDHS: Nepal Demographic and Health Survey

CI: Confidence Interval

[1]*Variable with p-value less than <0.2 from unadjusted model were included into multivariable analysis*

**p-value<0.05, **p-value<0.01, *** p-value<0.001*

Model 1 shows Crude Odds Ratio (COR), Model 2 shows interaction between place of residence and frequency of watching television, Model 3 shows Adjusted Odds Ratio (AOR)

Supplementary Table 2: Crude odds ratios for factors associated with overweight and obesity compared to normal weight for urban area among women of Nepal, NDHS 2016.

Variable	COR	Lower Limit	Upper Limit	AOR	Lower Limit	Upper Limit
**Age Group (in years)**						
15-24	Ref			Ref		
25-34	4.0***	3.3	4.8	2.3***	1.8	2.9
35-49	6.5***	5.4	7.8	3.9***	2.9	5.1
**Province of Residence**						
Province 1	Ref			Ref		
Province 2	0.4***	0.3	0.5	0.4***	0.3	0.5
Province 3	1.3[1]	0.9	1.9	1.0	0.8	1.4
Province 4	1.2	0.8	1.8	1.2	0.9	1.6
Province 5	0.7[1]	0.5	1.0	0.6	0.5	0.9
Province 6	0.5**	0.4	0.8	0.8	0.5	1.1
Province 7	0.3***	0.2	0.4	0.4***	0.3	0.5
**Ecological Zone of Residence**						
Mountain	Ref			Ref		
Hill	1.4	0.8	2.5			
The Terai	1.0	0.5	1.8			
**Highest Educational Status**						
No Formal Education	Ref			Ref		
Primary	1.2[1]	1.0	1.5	1.4**	1.2	1.8
Secondary	0.6***	0.5	0.8	1.3*	1.0	1.6
Higher	0.5***	0.4	0.7	1.1	0.8	1.4
**Currently employment**						
No	Ref			Ref		
Yes	1.3***	1.1	1.5	1.0	0.8	1.1
**Wealth index**						
Poorest	Ref			Ref		

Poorer	1.2[1]	0.9	1.6	1.2	0.9	1.6
Middle	1.6**	1.2	2.1	1.4*	1.1	2.0
Richer	2.4***	1.8	3.2	2.2***	1.6	3.0
Rich	5.0***	3.7	6.7	4.3***	3.0	6.1
**Marital Status**						
Single	Ref			Ref		
Married	5.9***	4.8	7.3	2.1***	1.5	3.0
Separated/ Divorced/ Widowed	4.5***	2.9	7.0	1.5	0.9	2.6
**Parity**						
0	Ref			Ref		
1	3.2***	2.5	4.1	1.3	0.9	1.8
2	5.9***	4.8	7.3	1.5**	1.1	2.2
3	5.9***	4.7	7.5	1.5	1.0	2.1
3+	4.8***	3.8	6.1	1.2	0.8	1.9
**Number of Household Member**						
≤5	Ref			Ref		
>5	0.7***	0.6	0.9	0.9	0.8	1.1
**Frequency of watching TV**						
Not at all	Ref			Ref		
Less than once a week	1.1	0.9	1.4	1.1	0.8	1.4
At least once a week	1.8***	1.5	2.2	1.4**	1.1	1.7

NDHS: Nepal Demographic and Health Survey

CI: Confidence Interval

AOR: Adjusted Odds Ratio

COR: Crude Odds Ratio

[1]*Variable with p-value less than <0.2 from unadjusted model were included into multivariable analysis*

**p-value<0.05, **p-value<0.01, *** p-value<0.001*

Supplementary Table 3: Adjusted odds ratios for factors associated with overweight and obesity compared to normal weight for rural area among women of Nepal, NDHS 2016.

**Variable**	COR	Lower Limit	Upper Limit	AOR	Lower Limit	Upper Limit
**Age Group (in years)**						
15-24	Ref			Ref		
25-34	3.4***	2.6	4.5	2.5***	1.7	3.5
35-49	4.1***	3.1	5.3	3.4***	2.3	5.1
**Province of Residence**						
Province 1	Ref			Ref		
Province 2	0.4***	0.2	0.6	0.3***	0.2	0.5
Province 3	1.6*	1.0	2.6	2.3***	1.4	3.6
Province 4	1.4[1]	0.9	2.2	1.5*	1.0	2.4
Province 5	0.7[1]	0.5	1.1	0.7	0.5	1.1
Province 6	0.3***	0.2	0.5	0.5*	0.3	0.9
Province 7	0.4***	0.2	0.6	0.4**	0.3	0.7
**Ecological Zone of Residence**						
Mountain	Ref			Not incorporated in the final model		
Hill	1.3	0.8	2.1			
The Terai	0.9	0.6	1.5			
**Highest Educational Status**						
No Formal Education	Ref			Ref		
Primary	1.3[1]	1.0	1.7	1.8***	1.3	2.4
Secondary	0.6**	0.5	0.8	1.4*	1.0	2.0
Higher	0.7[1]	0.5	1.0	1.2	0.8	2.0
**Currently employment**						
No	Ref			Ref		
Yes	1.3	1.0	1.7			
**Wealth index**						
Poorest	Ref			Ref		
Poorer	1.6**	1.2	2.2	1.5**	1.1	2.1

Middle	1.5*	1.0	2.0	1.7**	1.2	2.5
Richer	2.0***	1.4	2.9	2.2***	1.5	3.3
Rich	4.7***	2.8	7.8	5.0***	2.9	8.6
**Marital Status**						
Single	Ref			Ref		
Married	3.6***	2.6	5.0	1.8*	1.0	3.1
Separated/ Divorced/ Widowed	3.6***	2.0	6.7	1.4	0.6	3.0
**Parity**						
0	Ref			Ref		
1	1.7**	1.1	2.4	0.8	0.5	1.4
2	3.7***	2.6	5.2	1.3	0.7	2.1
3	4.3***	3.1	6.1	1.3	0.8	2.3
3+	3.2***	2.3	4.4	1.0	0.6	1.9
**Number of Household Member**						
≤5	Ref			Ref		
>5	0.7***	0.5	0.8	0.8	0.7	1.0
**Frequency of watching TV**						
Not at all	Ref			Ref		
Less than once a week	1.0	0.8	1.3	1.0	0.7	1.3
At least once a week	1.4**	1.1	1.9	1.1	0.8	1.5

NDHS: Nepal Demographic and Health Survey

CI: Confidence Interval

AOR: Adjusted Odds Ratio

COR: Crude Odds Ratio

[1]*Variable with p-value less than <0.2 from unadjusted model were included into multivariable analysis*

**p-value<0.05, **p-value<0.01, *** p-value<0.001*

Supplementary Table 4: Crude and Adjusted odds ratios for factors associated with overweight and obesity (using traditional BMI-cutoff) compared to normal weight among women of Nepal, NDHS 2016.

Variable	Model 1			Model 2			Model 3		
	OR	Lower Limit	Upper Limit	OR	Lower Limit	Upper Limit	OR	Lower Limit	Upper Limit
**Age Group (in years)**									
15-24	Ref						Ref		
25-34	5.8	4.7	7.2				3.1	2.4	4.0
35-49	8.6	7.0	10.6				4.9	3.7	6.6
**Place of Residence**									
Rural	Ref			Ref			Ref		
Urban	1.8	1.4	2.3	1.1	0.8	1.4	0.9	0.7	1.3
**Province of Residence**									
Province 1	Ref						Ref		
Province 2	0.3	0.2	0.5				0.4	0.3	0.5
Province 3	1.3	0.9	1.8				1.0	0.8	1.3
Province 4	1.1	0.8	1.6				1.0	0.8	1.3
Province 5	0.6	0.4	0.9				0.6	0.5	0.8
Province 6	0.3	0.2	0.5				0.5	0.4	0.7
Province 7	0.2	0.1	0.3				0.3	0.2	0.4
**Ecological Zone of Residence**									
Mountain	Ref						Ref		
Hill	1.6	1.0	2.5				0.8	0.6	1.1

The Terai	1.3	0.8	2.0		0.6	0.5	0.9
**Highest Educational Status**							
No Formal Education	Ref				Ref		
Primary	1.2	1.0	1.5		1.4	1.2	1.8
Secondary	0.6	0.5	0.8		1.2	1.0	1.5
Higher	0.6	0.5	0.8		1.1	0.9	1.5
**Currently employment**							
No	Ref				Ref		
Yes	1.2	1.1	1.4		0.9	0.8	1.0
**Wealth index**							
Poorest	Ref				Ref		
Poorer	1.9	1.5	2.6		1.9	1.4	2.5
Middle	2.1	1.6	2.7		2.0	1.5	2.7
Richer	3.5	2.6	4.6		3.1	2.3	4.2
Rich	8.0	6.1	10.6		7.1	5.1	9.8
**Marital Status**							
Single	Ref				Ref		
Married	9.1	6.9	12.0		3.1	2.1	4.7
Separated/ Divorced/ Widowed	8.0	5.0	12.7		2.5	1.5	4.4
**Parity**							
0	Ref				Ref		

1	4.0	3.1	5.2				1.1	0.8	1.6
2	7.7	6.1	9.8				1.3	0.9	1.8
3	7.3	5.6	9.4				1.2	0.8	1.7
3+	5.8	4.4	7.5				1.0	0.7	1.5
**Number of Household Member**									
≤5	Ref						Ref		
>5	0.7	0.6	0.8				0.9	0.7	1.0
**Frequency of watching TV**									
Not at all	Ref			Ref			Ref		
Less than once a week	1.3	1.1	1.7	1.3	0.9	1.8	1.2	0.8	1.7
At least once a week	2.3	1.9	2.8	2.3	1.7	3.0	1.4	1.0	1.9
**Interaction Between Place of Residence with Frequency of Watching TV**									
Urban Residence × Not at all watches TV				Ref			Ref		
Urban Residence × Watches TV less than once a week				1.2	0.8	1.8	1.1	0.7	1.7
Urban Residence × Watches TV at least once a week				1.4	1.0	2.0	1.1	0.7	1.6

BMI: Body Mass Index

NDHS: Nepal Demographic and Health Survey

CI: Confidence Interval

[1]*Variable with p-value less than <0.2 from unadjusted model were included into multivariable analysis*

**p-value<0.05, **p-value<0.01, *** p-value<0.001*

Model 1 shows Crude Odds Ratio (COR), Model 2 shows interaction between place of residence and frequency of watching television, Model 3 shows Adjusted Odds Ratio (AOR)

Supplementary Table 5: Crude odds ratios for factors associated with overweight and obesity (using traditional BMI-cutoff) compared to normal weight for urban area among women of Nepal, NDHS 2016.

Variable	COR	Lower Limit	Upper Limit	AOR	Lower Limit	Upper Limit
**Age Group (in years)**						
15-24	Ref			Ref		
25-34	6.2	4.8	8.0	3.1	2.3	4.3
35-49	9.9	7.7	12.7	5.0	3.6	7.1
**Province of Residence**						
Province 1	Ref			Ref		
Province 2	0.4	0.2	0.6	0.4	0.3	0.6
Province 3	1.3	0.9	2.0	0.9	0.6	1.2
Province 4	1.2	0.8	1.9	1.0	0.7	1.4
Province 5	0.6	0.4	0.9	0.6	0.4	0.8
Province 6	0.4	0.3	0.7	0.6	0.4	0.9
Province 7	0.2	0.1	0.3	0.3	0.2	0.4
**Ecological Zone of Residence**						
Mountain	Ref			Ref		
Hill	1.6	0.7	3.2	1.0	0.6	1.5
The Terai	1.0	0.5	2.2	0.7	0.5	1.2
**Highest Educational Status**						
No Formal Education	Ref			Ref		
Primary	1.2	0.9	1.6	1.4	1.1	1.8
Secondary	0.6	0.5	0.8	1.2	0.9	1.5
Higher	0.5	0.4	0.7	1.1	0.8	1.5
**Currently Employed**						
No	Ref			Ref		
Yes	1.3	1.1	1.5	0.9	0.7	1.0
**Wealth Index**						
Poorest	Ref			Ref		
Poorer	1.8	1.2	2.7	1.7	1.1	2.5
Middle	2.1	1.4	3.1	1.8	1.2	2.8

Richer	3.6	2.4	5.3	2.9	1.9	4.3
Rich	8.1	5.5	11.9	6.7	4.4	10.2
**Marital Status**						
Single	Ref			Ref		
Married	10.6	7.6	14.6	3.2	2.0	5.2
Separated/Divorced/Widowed	6.8	3.8	12.1	2.2	1.1	4.3
**Parity**						
0	Ref			Ref		
1	5.0	3.6	6.8	1.3	0.8	2.0
2	8.7	6.5	11.4	1.3	0.9	2.0
3	7.8	5.7	10.7	1.2	0.8	1.9
3+	7.1	5.2	9.8	1.2	0.7	1.9
**Number of Household Members**						
≤5	Ref			Ref		
>5	0.7	0.6	0.9	0.9	0.8	1.1
**Frequency of Watching TV**						
Not at All	Ref			Ref		
Less Than Once a Week	1.5	1.1	2.0	1.4	1.0	1.9
At Least Once a Week	2.5	2.0	3.3	1.7	1.3	2.2

BMI: Body Mass Index

NDHS: Nepal Demographic and Health Survey

CI: Confidence Interval

AOR: Adjusted Odds Ratio

COR: Crude Odds Ratio

[1]*Variable with p-value less than <0.2 from unadjusted model were included into multivariable analysis*

**p-value<0.05, **p-value<0.01, *** p-value<0.001*

Supplementary Table 6: Crude odds ratios for factors associated with overweight and obesity (using traditional BMI-cutoff) compared to normal weight for rural area among women of Nepal, NDHS 2016.

Variable	COR	Lower Limit	Upper Limit	AOR	Lower Limit	Upper Limit
**Age Group (in years)**						
15-24	Ref			Ref		
25-34	4.9	3.3	7.2	3.2	2.0	5.1
35-49	6.1	4.2	9.0	4.9	2.9	8.4
**Province of Residence**						
Province 1	Ref			Ref		
Province 2	0.3	0.2	0.5	0.2	0.1	0.4
Province 3	1.0	0.6	1.7	1.5	0.9	2.4
Province 4	1.0	0.6	1.7	1.2	0.8	1.9
Province 5	0.6	0.4	1.1	0.6	0.4	1.0
Province 6	0.2	0.1	0.3	0.4	0.2	0.7
Province 7	0.3	0.1	0.5	0.3	0.2	0.6
**Ecological Zone of Residence**						
Mountain	Ref			Not incorporated in the final model		
Hill	1.1	0.6	2.0			
The Terai	1.1	0.6	2.1			
**Highest Educational Status**						
No Formal Education	Ref			Ref		
Primary	1.1	0.8	1.6	1.5	1.0	2.2
Secondary	0.6	0.5	0.9	1.4	1.0	2.1
Higher	0.8	0.5	1.2	1.3	0.8	2.3
**Currently Employed**						
No	Ref			Ref		
Yes	1.2	0.9	1.7	1.0	0.7	1.3
**Wealth Index**						
Poorest	Ref			Ref		
Poorer	2.1	1.5	3.1	1.9	1.3	2.8
Middle	2.0	1.3	3.0	2.2	1.4	3.4

Richer	3.1	2.0	4.9	3.1	2.0	5.0
Rich	7.5	4.2	13.4	7.2	3.9	13.3
**Marital Status**						
Single	Ref			Ref		
Married	5.9	3.5	9.9	2.4	1.1	5.1
Separated/Divorced/Widowed	9.2	4.2	20.3	2.8	1.1	7.3
**Parity**						
0	Ref			Ref		
1	2.1	1.3	3.6	0.7	0.4	1.5
2	5.3	3.3	8.3	1.2	0.6	2.3
3	5.8	3.6	9.1	1.2	0.6	2.3
3+	3.6	2.3	5.7	0.8	0.4	1.6
**Number of Household Members**						
≤5	Ref			Ref		
>5	0.6	0.4	0.8	0.7	0.6	1.0
**Frequency of Watching TV**						
Not at All	Ref			Ref		
Less Than Once a Week	1.1	0.8	1.6	1.0	0.7	1.5
At Least Once a Week	1.7	1.2	2.4	1.2	0.8	1.7

BMI: Body Mass Index

NDHS: Nepal Demographic and Health Survey

CI: Confidence Interval

AOR: Adjusted Odds Ratio

COR: Crude Odds Ratio

[1]*Variable with p-value less than <0.2 from unadjusted model were included into multivariable analysis*

**p-value<0.05, **p-value<0.01, *** p-value<0.0001*
