# Supplementary material for: Association between the frequency of television watching and overweight and obesity among women of reproductive age in Nepal: Analysis of data from the Nepal Demographic and Health Survey 2016
Source: PLoS One. 2020 Feb 10;15(2):e0228862. doi: 10.1371/journal.pone.0228862 (PMC7010261; doi:10.1371/journal.pone.0228862)
Supplement: Attachment — Submitted filename: Response to Reviewers.docx [file pone.0228862.s002.docx]

**Submitted Title:** Association between the frequency of television watching and overweight and obesity among women of reproductive age in Nepal: Analysis of data from the Nepal Demographic and Health Survey 2016

**Reference No:** PONE-D-19-20505

**Additional Editor Comments (if provided):**
*In general, the manuscript needs some careful reading to improve use of English, which is pretty good, but nevertheless there are some errors (e.g. overuse of ‘the’).*

**Response:** Thanks! We have revised the manuscript. The language of the manuscript was further edited by an in-house editor who is a native English speaker.

*Abstract In general, the method needs to be more concise (e.g. need to summarise, not list covariates here), also you need to mention the co-variates in the results.*

**Response:** Thanks for this important comment! We have removed the list of covariates from the abstract. We have also added the summary of the covariates in the results.

*Introduction
ln 74: give % increase for men. Otherwise fine*

**Response:** Thanks! We have added the percentage increase for men as per suggestion.

*Method
Ln 105: is there a reference for the probability proportional to size method?*

**Response:** Thanks! We have added the reference. It is the Nepal Demographic and Health Survey 2016 report.

*Ln 133: what are ' 'The categories''? this needs more explanation*

**Response:** Thanks! We have revised the sentence: “The categories of the covariates are mentioned in Table 1.”

*Ln 130: it is not clear how the literature review led to identification of the co-variates*

**Response:** Thanks! In previous studies these covariates were found to be associated with overweight and obesity. So we have included them in the analysis. We have mentioned that in the revised manuscript and have added the reference.

*Ln 133-135: why are the selected assets relevant to this study?*

**Response:** Thanks! The selected assets were used to generate household wealth index which was then divided into quintiles to generate household wealth status, which is a covariate in our study. We have revised that paragraph as following: “The NDHS 2016 collected data on selected assets, such as household construction materials, types of water source and sanitation facilities, electricity and other belongings (e.g., television, bicycle, etc.). Principal component analysis was then conducted to measure household wealth index [16,18,19]. The wealth index was further divided into quintiles to generate household wealth status.”

*Results
I don’t think just reporting the highest value in each category gives a clear picture of the sample*

**Response:** Thanks! We have added additional information.

*Figures not clear*

**Response:** Thanks! We have removed both the figures.

*Ln 192-194: it is not meaningful to the reader to discuss Province No 3 and the Hill region without more description of context.*

**Response:** Thanks! We have added a paragraph called study settings under methods section where we discussed about the context including province and ecologic zone.

*Think about how best to present your results to make them coherent e.g. urban vs rual might go first?*

**Response:** Thanks! We have reorganized the findings. First we presented the odds ratio in the urban area, then rural area. At last we presented the overall odds ratio.  *Discussion
p 238 Province and Hill need more explanation/context as before.*

**Response:** Thanks! We have added a paragraph called study settings under methods section where we discussed about the context including province and ecologic zone.

*Reviewer #1: ASSOCIATION BETWEEN THE FREQUENCY OF TELEVISION WATCHING AND OVERWEIGHT AND OBESITY AMONG WOMEN OF REPRODUCTIVE AGE IN NEPAL: ANALYSIS OF DATA FROM THE NEPAL DEMOGRAPHIC AND HEALTH SURVET 2016


OVERALL COMMENT:
This manuscript looks for the association between TV watching and overweight and obesity. The finding is not novel and several previous papers have shown such association in LMIC and Asian countries. Moreover, there is a concern related to the use of ordered logistic regression as the model to look for the association of interest, instead of using multinomial logistic regression. Such concern is increased as OR usually overestimates strength of the association when the outcome is highly prevalent (more than 10%) as in the case of overweight or obesity. A potential option would be to use prevalence ratios, but in this case using two different models (i.e. comparing overweight vs. normal and obesity vs. normal). Finally, why the authors use Asian cut-offs for BMI is not well explained. Ideally, I would like to see the association with traditional BMI cut-off as sensitivity analysis.*

**Response:** Many thanks for the comments. Thanks for this important suggestion. However, we believe that ordered logistic regression is the best way, because the outcome variable is an ordinal variable. Previous studies from this region also used ordered logistic regression.

India: <https://journals.plos.org/plosone/article?id=10.1371/journal.pone.0221758>

Myanmar: <https://bmjopen.bmj.com/content/9/3/e024680>

Thanks! We have added the reason for using Asian BMI cut-offs: “Asia-specific cutoff was used because it was advised by the World Health Organization (WHO) expert consultation group, due to different associations BMI, body fat with health risks compared to the European population [17].” We have also added analysis the traditional cut-offs, which reveal similar findings like the Asian cut off.  *MAJOR COMMENTS:
Abstract:
- The direction of the association is capital here... is that obese women are more prone to watch TV? Or is that watching TV associated with overweight/obesity? Please define appropriately in the abstract.*

**Response:** Thanks for this comment. We have edited accordingly.

*- Reasons for using Asian BMI cut-offs should be clearly explained as they are not the usual cut-offs... in addition, please include sensitivity analysis using traditional cut-offs*

**Response:** Thanks! We have added the reason for using Asian BMI cut-offs: “Asia-specific cutoff was used because it was advised by the World Health Organization (WHO) expert consultation group, due to different associations BMI, body fat with health risks compared to the European population [17].” We have also added analysis the traditional cut-offs.

*- What proportion of people watchs TV at least once a week? Is that proportion important?*

**Response:** Thanks! We have mention in the findings section: “Around half of the respondents (50.6%) reported watching television at least once a week, whereas more than one-fourth (28.7%) of them watched television not at all.

The proportion of television watchers at least once a week was significantly higher in the urban area compared to the rural area (urban: 60.0% vs. rural: 34.5%, *p*-value<0.0001).” This proportion is important to understand the basic demographic characteristics of the respondents.

*Introduction:
- Please define appropriately the direction of the association of interest throughout the text.*

**Response:** Thanks for this comment. We have edited accordingly.  *Methods:
- Line 102: randomized sampling or random sampling? This is not a trial.*

**Response:** Thanks! We have edited it to random sampling.

*- How the individual was selected for the study? Were all the women from household selected? Only one? Please explain.*

**Response:** Thanks! We have added a sentence: “All women aged 15-49 years, who were either the permanent residents of the households or stayed at the household night before the survey were interviewed.”

*- Line 118: Is it sex a variable? But all are women or not?*

**Response:** Thanks! We have deleted the word ‘sex’.

*- Line 124: Reasons for using Asian BMI cut-offs should be detailed... In addition, a sensitivity analysis is needed to compare results with traditional cut-offs*

**Response:** Thanks! We have added the reason for using Asian BMI cut-offs: “Asia-specific cutoff was used because it was advised by the World Health Organization (WHO) expert consultation group, due to different associations BMI, body fat with health risks compared to the European population [17].” We have also added analysis the traditional cut-offs.

*- Not sure if ordered logistic regression is the best way to create the model to look for the association of interest, instead of using multinomial logistic regression for example. Such concern is increased as OR usually overestimates strength of the association when the outcome is highly prevalent (more than 10%) as in the case of overweight or obesity. A potential option should be to use prevalence ratios, but in this case using two different models (i.e. comparing overweight vs. normal and obesity vs. normal).*

**Response:** Thanks for this important suggestion. However, we believe that ordered logistic regression is the best way, because the outcome variable is an ordinal variable. Previous studies from this region also used ordered logistic regression.

India: <https://journals.plos.org/plosone/article?id=10.1371/journal.pone.0221758>

Myanmar: <https://bmjopen.bmj.com/content/9/3/e024680>

*- Line 147-148: This is an exploratory model looking for potential variables independently associated with an outcome; but according to the objectives the idea sounds more like a confirmatory model (when you show your crude model and then the adjusted model controlling for potential confounders)*

**Response:** Thanks for this comment.

*- Line 155: re collinearity: sounds like a result more than a part of the methods.*

**Response:** Thanks! We the statement in the findings section.

*- Line 155-157: how interaction was assessed?*

**Response:** We have shown the interaction in Model 2 under the supplementary file 1.

*Findings
- Line 168: How the number 6031 was obtained? A flowchart may be useful here instead of the pie figure (this does not give more information than text).*

**Response:** Thanks! we have added a flow chart (Figure 1).

*- A sensitivity analysis is needed using traditional BMI cut-offs instead of Asian cut-offs... is the association found the same? Is it significant?*

**Response:** Thanks! We have added analysis the traditional cut-offs. *Discussion
- Please discuss about the relevance of the findings... how are they important for the public health perspective?*

**Response:** Thanks! We have mentioned this in the discussion section: “The higher burden of overweight and obesity is a public health problem in Nepal, which plays a role in the increasing burden of NCDs in the country. Currently, nearly three-fifths of the total disease burden is attributable to NCDs [35]. The health system of Nepal is more focused on the curative aspects of NCDs, rather than the preventive side [35]. Preventive programs should be strengthened in order to reduce the burden of overweight and obesity and thus ultimately reducing the overall burden of NCDs. Health promotion programs focusing on women of reproductive age should incorporate the importance of physical activity and less ‘sitting time’ for television watching, especially in the urban area. Future research should also include men and adolescents using a nationally representative sample to assess whether this association exists in those populations.”
