# Supplementary material for: Association between the frequency of television watching and overweight and obesity among women of reproductive age in Nepal: Analysis of data from the Nepal Demographic and Health Survey 2016
Source: PLoS One. 2020 Feb 10;15(2):e0228862. doi: 10.1371/journal.pone.0228862 (PMC7010261; doi:10.1371/journal.pone.0228862)
Supplement: Attachment — Submitted filename: Response to Reviewer.docx [file pone.0228862.s003.docx]

**Manuscript ID:** PONE-D-19-20505R1

**Submitted Title:** Association between the frequency of television watching and overweight and obesity among women of reproductive age in Nepal: Analysis of data from the Nepal Demographic and Health Survey 2016

Additional Editor Comments (if provided):

The manuscript is looking good now. Just some very minor points to attend to

Abstract
*ln 33 could “in the South Asian setting” just be “in South Asia”?*

**Response to Reviewer:** Revised as per advice.
*Ln 54 to 55 – should it read “A majority of the study participants was aged between 15 and 24 years (36.5%), AND resided in an urban area (63.2%), Province No. 3 (22.3%), and the Terai ecological region (49.5%).”*

**Response to Reviewer:** Revised as per advice.
*Ln 55-56 tense is wrong*

**Response to Reviewer:** Revised as per advice.
*Ln 58 one fourth should be a quarter*

**Response to Reviewer:** Revised as per advice.

Introduction
*Ln 84-85 “During the same time period, the global prevalence of women with obesity has increased by almost 465%, from 69 million to 390 million [6]” Edit suggested*

**Response to Reviewer:** Revised as per advice.
*Ln 105 (NDHS) 2016 does not align with the abstract (NDHS 2016) and elsewhere. Please make use of acronyms consistent*

**Response to Reviewer:** Revised as per advice.

Method
*Ln 108 Title should be Study Setting*

**Response to Reviewer:** Revised as per advice.
*Ln 125 – who are NEW ERA please describe*

**Response to Reviewer:** We have added: “NEW ERA is a non-profit, non-governmental research organization in Nepal.”
*Ln 142-143 to collect socio-demographic information Edit suggested*

**Response to Reviewer:** Revised as per advice.
*Ln 145 SECA scales needs more information e.g. manufacturer*

**Response to Reviewer:** Thank you for the suggestion! Unfortunately, the information was not provided in the NDHS 2016 final report.
*Ln 168 the findings using a traditional BMI cutoff were Edit suggested*

**Response to Reviewer:** Revised as per advice.
*In table 1 – I don’t think you need to keep repeating of the respondents or of the study participants? Also please be consistent don’t call them respondents and participants*

**Response to Reviewer:** Thank you for this important comment. We have deleted the repeating of the respondents or of the study participants. We now consistently mention them as ‘study participants’.
*Ln 181 NDHS 2016 data – be consistent*

**Response to Reviewer:** Revised as per advice.
*Ln 196 The NDHS 2016 edit suggested. Also is it one or two boards – please make this clear*

**Response to Reviewer:** Revised as per advice. Its two board. We have clarified this in the revised manuscript as: “The NDHS 2016 protocol was reviewed and approved by the ethical review board of the Nepal Research Council as well as the institutional review board of ICF International.”

Findings
*ln 208 “were” is missing*

**Response to Reviewer:** Revised as per advice.
*Ln 210 should this be and/or the Tari region*

**Response to Reviewer:** No. This will be and the Terai region.
*Ln 213 should be had fewer than*

**Response to Reviewer:** Revised as per advice.
*Ln 215 a quarter*

**Response to Reviewer:** Revised as per advice.
*Please be consistent – are they respondents or study participants? Also you don’t need to keep repeating this*

**Response to Reviewer:** Revised as per advice.
*Ln 232-235 Women who attained primary education only had the highest prevalence of overweight and obesity (p-value <0.001). The prevalence of overweight and obesity significantly increased with wealth index and nearly three-fifths of the women from the richest quintiles were overweight (30.1%) or obese (27.7%). THIS SEEMS COUNTERINTUITIVE – DO YOU DISCUSS IT? (I would have thought wealthy women would be better educated?) Also is it not coherent with the discussion which says (ln274-276 The prevalence of overweight and obesity was higher among women who were older, residing in Province No. 3, residing in the Hill region, had higher educational attainment, and belonged to the richest wealth quintiles.*

**Response to Reviewer:** Thanks! We have revised that. In the logistics regression (inS1 File), all the educational group had higher odds of being overweight and obese compared to those who did not receive formal education.

Discussion
*Ln 264 between frequency of television Suggested edit*

**Response to Reviewer:** Revised as per advice.
*Ln 270 Nepal were either Suggested edit*

**Response to Reviewer:** Revised as per advice.
*Ln 288 A study of Nepalese Suggested edit*

**Response to Reviewer:** Revised as per advice.
*Ln 291 in Western countries Suggested edit*

**Response to Reviewer:** Revised as per advice.
*Ln 292-293 In this study, the association between watching television once a week and overweight and obesity was significant in the urban area, but not in the rural area. Suggested edit*

**Response to Reviewer:** Revised as per advice.
*Ln 304 This may be one reason Suggested edit*

**Response to Reviewer:** Revised as per advice.
*Ln 309 – should you spell out non-communicable diseases the first time?*

**Response to Reviewer:** We have spelled it out in the introduction.
*Ln 318-319 . First, a nationally representative sample was used for this study and therefore the findings of this study are generalizable to the target population. Suggested edit*

**Response to Reviewer:** Revised as per advice.

Conclusion
*Ln 331 The high burden of overweight and obesity in Nepal is a public health problem that warrants immediate action. Suggested edit*

**Response to Reviewer:** Revised as per advice.
*Ln 333 age living in urban areas Suggested edit*

**Response to Reviewer:** Revised as per advice.
*Check the Figure – there is a line spacing issue in the second last box*

**Response to Reviewer:** Revised as per advice.

**Reviewer #1:**

*All the comments have been addressed. However, two minor points appear:
- Introduction: line 85, 69 million or 390 million are not prevalence as stated, they are absolute numbers.*

**Response to Reviewer:** Edited as per advice.
*- Outcome variables and covariates: line 170: should be "and <30 kg/m2" to avoid overlap with the obesity category.*

**Response to Reviewer:** Edited as per advice.
